# Edge detection using fast pixel based matching and contours mapping algorithms

**DOI:** 10.1371/journal.pone.0289823

**Published:** 2023-08-11

**Authors:** T. S. Arulananth, P. Chinnasamy, J. Chinna Babu, Ajmeera Kiran, J. Hemalatha, Mohamed Abbas

**Affiliations:** 1 Department of Electronics and Communication Engineering, MLR Institute of Technology, Hyderabad, Telangana, India; 2 Department of Computer Science and Engineering, MLR Institute of Technology, Hyderabad, Telangana, India; 3 Department of Electronics and Communication Engineering, Annamacharya Institute of Technology and Sciences, Rajampet, Andhra Pradesh, India; 4 Department of CSE, AAA College of Engineering and Technology, Amathur, Tamilnadu, India; 5 Electrical Engineering Department, College of Engineering, King Khalid University, Abha, Saudi Arabia; Wuhan University of Science and Technology, CHINA

## Abstract

Current methods of edge identification were constrained by issues like lighting changes, position disparity, colour changes, and gesture variability, among others. The aforementioned modifications have a significant impact, especially on scaled factors like temporal delay, gradient data, effectiveness in noise, translation, and qualifying edge outlines. It is obvious that an image’s borders hold the majority of the shape data. Reducing the amount of time it takes for image identification, increase gradient knowledge of the image, improving efficiency in high noise environments, and pinpointing the precise location of an image are some potential obstacles in recognizing edges. the boundaries of an image stronger and more apparent locate those borders in the image initially, sharpening it by removing any extraneous detail with the use of the proper filters, followed by enhancing the edge-containing areas. The processes involved in recognizing edges are filtering, boosting, recognizing, and localizing. Numerous approaches have been suggested for the previously outlined identification of edges procedures. Edge detection using Fast pixel-based matching and contours mappingmethods are used to overcome the aforementioned restrictions for better picture recognition. In this article, we are introducing the Fast Pixel based matching and contours mapping algorithms to compare the edges in reference and targeted frames using mask-propagation and non-local techniques. Our system resists significant item visual fluctuation as well as copes with obstructions because we incorporate input from both the first and prior frames Improvement in performance in proposed system is discussed in result section, evidences are tabulated and sketched. Mainly detection probabilities and detection time is remarkably reinforced Effective identification of such things were widely useful in fingerprint comparison, medical diagnostics, Smart Cities, production, Cyber Physical Systems, incorporating Artificial Intelligence, and license plate recognition are conceivable applications of this suggested work.

## Introduction

For edge detection in an image, the Prewitt operator is utilized. It recognizes Horizontal and Vertical Edges, two different sorts of edges [1, 2]. It is determined using the difference between the intensities of the relevant pixels in an image. Derivative masks refer to any mask that is utilized for edge detection. Because an image is also a signal, as we have often explained in this tutorial series, only differentiating allows for the calculation of signal modifications. Due of these kinds of operators are also referred to as hypothetical operations or dynamic filters. The following characteristics ought to be shared by all derivative masks:

- The mask must have the opposite sign
- The sum of the mask must be zero
- More mass equals more edge

When applied, this mask gives the photo its sharp vertical boundaries. Like a first-order derivate, it solely determines the variation in pixel illumination in an edge section [3]. Since the middle column is zero, the calculation only takes into account the discrepancy among the opposite right and left pixel numbers along that edge. Consequently, compared to the original appearance the edge brightness has been enhanced and improved. The masking device will only pick up edges in the direction that’s horizontal because of how the zeros section is oriented. The image’s horizontally limits will be plainly visible once you place this mask on it. Hence, we should develop a system with new hardware and which must to overcome the above limitations. Implement the hardware for the fulfillment of the above-mentioned objectives in effective manner [4, 5]. This specific hardware is implemented on the Digital signal processors and FPGA kits using the suitable software tools. It is essentials for the image processing environment. FPGA and DSP processors having the capabilities of implanting image processing features in it. Two new methodologies are namely fast pixel-based matching and contours mapping algorithms introduced for the betterment from the above problems. These methods are different from the traditional edge detection techniques [5]. Some of the existing systems have the limitations like high computational cost and other methods leads to poor performance. Edges and boundaries are giving a genuine parameter of the face. Important features of the face can be extracted from the edges with maximum detection probabilities. Critical scenarios in image processing environment can be handled by an effective hardware [16].

In an image processing environment time delay is take an important credential. Desirable to achieve the above-mentioned quality parameter, here proposed some approaches is known as fast pixel based matching contours mapping algorithms [6]. These methods are different from the traditional edge detection techniques.

We design our model based on mask-propagation to keep efficiency, where the non-local structure [9] adopt to generate the object mask using the obtained target object’s appearance information. Specifically, we design video object segmentation model called Fast Pixel-Matching network with Contours mapping algorithms, which includes a newly designed pixel-matching module and a channel attention module. The pixel-matching module is designed to match pixels between the target frame and the reference frames with given ground-truth mask or estimated mask. The channel attention module is used to augment the matched feature map to achieve better decoding. Extensive experiments have shown that our network can achieve a new state-of-the-art performance without loss of efficiency. Our main contribution is summarized as follows:

- We offer a video object segmentation model that successfully balances both rapidity and precision. To reduce computing demands, the algorithm is not dependent on online fine-tuning approaches, but it can nevertheless adaptively detect changes in the intended object’s aspect via both picture and anticipated mask data taken from the frame preceding it.
- By combining data from multiple frames, our suggested non-local pixel-matching module may successfully forecast the desired object disguise. By visualizing the acquired mappings of features, the suggested methodology additionally offers high level comprehensibility
- Using the same research setup, our framework obtains new outstanding results in terms of MSR, NSR and SIM values with various algorithms.

## Literature survey

Giuseppe Papari and colleagues [1], a list of numerous edge- and line-oriented contour recognition methods was provided. That has been proposed during the past 20 years. It is possible to discern between edges and contours. There are two types of contour detection systems: local and global processors. They conclude that contour recognition has advanced to a high level of elegance in light of multifaceted contour a description (by changes in luminance, colour, or texture), techniques for reducing the impact of noise and substance on contour concealment, sensory organization, multi-scale reasons, and high-level perception data.

Jiangping Wang et al. [2], Described about the vast range of applications it has in computer vision and multimedia retrieval, an effective and efficient image contour detector is greatly needed. They investigate the connection between edge detection and contour detection in this work, and a proposed edge-based picture contour detection technique is based on their findings. For efficiency, this method completely utilizes simple edge information. The suggested contour detector operates substantially more quickly than current state-of-the-art algorithms while maintaining excellent accuracy, making it appropriate for use in large-scale applications, according to testing on benchmark data sets.

Syed Mohammad Abid et al. [3], Edge detection in a depth image plays a significant part in computer vision applications. Illustrated 3D measurement methods are widely employed in the manufacturing sector. In this research, proposed an edge identification method for depth images based on morphological and image-based smoothing techniques. Applied the Median filtering principle, which is known for its edge preservation capabilities, in their method.

Jonghoon Seo et al. [4], Outlined about Objects are distinguished from the backdrop using contour pixels. Because they are straightforward and effective for object detection, contour pixels are commonly employed for smart/wearable image sensor systems. The suggested system categorizes the different sorts of contour pixels according to their local pattern. We compare the recommended algorithm’s execution efficiency and precision to that of traditional approaches. In the results of the experiments, the suggested algorithm outperforms the competition. Furthermore, it is capable of delivering compressed contour pixel data and accurately recovering every single one of them, particularly the inner-outer edge, which is impossible to restore using conventional techniques.

Anisotropic Directional Derivative (ANDD) interpretations are introduced by Shui PL et al. [5] as a corner detection and classification. Depending on the orientated angle, the ANDD encoding at a pixel defines the local directional grayscale difference close to the pixel. The suggested corner detector combines the principles of intensity- and contour-based detection. It contains three tumbling sections. The smart detection system initially acquires the edge map of an image, from which contour are subsequently retrieved and reconstructed. The ANDD version is then computed and its highest intensity at each pixel on the contours is used to normalize it. The proposed classifier is capable of discriminating between Y-type edges, higher order edges, and simple squares.

W. Zhang et al. [6] Demonstrate how corner detection is used in a variety of statistical image analysis and learning applications, including recognizing items and image comparison. According to our research, the accuracy with which current corner recognition algorithms are able to differentiate between fringes and corners results in inaccurate corner findings. This article evaluates the reduction of Gaussian noise by Gaussian directed differential screens.

Peng-Lang Shui and coworkers [7], Conventional distinction-based edge detection encounters a rapid effectiveness drop when communicated regarding impulse sounds alter images. Evolutionary processors like median filtration and modified median filtration are capable of eliminating impulse distortion. The outcomes demonstrate that, with impulse noise cases demonstrating the greatest performance.

Mafi M et al. [8], Has researched and presented a reliable edge detection technique that makes use of a combined approach to de-noising images. This technique has been shown to be able to survive impulsive noise, sometimes known as salty and spicy noise, particularly at high concentrations. Using measurements for significant correlation, structural comparable index, and maximum signal to noise proportion, we show that the recommended switching adaptive median and fixed weighted mean filter (SAMFWMF) provides the greatest edge detection and edge feature conservation.

Pritamdas K et al. [9], window containing the discovered noisy pixel is then taken into further consideration, and the pixels inside it are given exponential weights based on how similar they are to their other nearby neighbors, both spatially and radio metrically. The biased normal of the pixels inside the window is then used to replace the noisy pixels [10–12].

Wang et al. [13], introduced the new study that examines four phases. The procedure, constructed around the characteristic point approach, is used by the front-end graphical odometer to acquire and correlate properties across pictures and solve movement of the camera across consecutive images iteratively using the closest possible location approach.

Verykokou and Ioannidis [14], provided a thorough discussion of various 3D modelling techniques that can be used to produce three-dimensional reconstructions of the exterior or interior surfaces of various target types. In the present setting, it tackles the issue of producing 3D models from scans and covers the issues of 3D modelling utilizing photos using various approaches, an exhaustive list of three-dimensional scanners, and the basic principles of operation of every kind of scanning. Finally, it describes a few 3D modelling applications that go beyond the well-known geographic aspects.

Li et al. [15], suggested the solution by deep learning-based approach and standard operators like HDE and Sobel are combined in an edge feature extraction method to categorise and access images more accurately. This reduces the quantity of data needed to run deep learning-based algorithms, achieves model adaptability, improves categorization and retrieval accuracy, and compresses the data. Benchmarking data sets are used to achieve all these superior outcomes. Thus, by suggesting a unique approach, all of these are accomplished. Santhanam et al. [16] provided evidence that the finely labelled contours can aid downstream applications of computer vision, including reconstructing three dimensions from a two-dimensional picture.

Cai et al. [17] results from the experiment and analytical findings show how the suggested artificial ecosystem technique creates the edge picture and how it can successfully address image recognition of edges issues. Kong et al. [18], The findings demonstrate that the suggested approach can detect more precisely defined framework edges as well as recognise full-field movement and mode form of constructions despite the need to set up artificial seeks on the framework beforehand, providing important information for assessing its skeletal circumstance, particularly for constructions that experience minuscule-amplitude energy.

Qian et al. [19], Ground acoustic tests have been carried out to confirm the suggested approach. First, the use of a solar wing construction led to the conclusion that the Digital Images Coherence technique for the signal acquisition was very accurate and feasible. Furthermore, the proposed approach was tested using an ultralow-frequency extensible cantilevered beam framework, which the theoretical baseline frequency that was 0.185 Hz, which also complied with the anticipated resolution. Experiments demonstrate that the Digital Image Coupling approach can efficiently assess the ultralow-frequency flexibility framework’s responsive response and determine its dynamical properties.

Peroš et al. [20], they suggested in this work to measure removals and track the behaviour of structural components under steady loads in real-world settings. For the computation of estimated removals from RGB+D pictures, removals acquired from a computer simulation were utilised as a guide, along with observations from an accurate linear variation differentially transformers (LVDT) sensors.

Yifan et al. [21], proposed a joint end-to-end segmentation of lines detection method based on Transformations, which is free from post-processing and optimization algorithms-guided intermediary filtering (edge/junction/region detection). By eschewing conventional algorithmic approaches for the edge component identification and grouping of perceptions operations, their approach, dubbed LinE element TRansformers (LETR), makes use of Transformers’ built-in designated queries, self-awareness process, and encoding-decoding methodology. We provide Transformers with a multi-scale encoder/decoder technique so they can recognise line segments with great precision even when there is a straight terminal proximity reduction.

Kong et al. [18], we provided a novel visual enhancement approach for an extremely small iris image. Searching for a collection of clipping limit values is done using the CPSSA. Then CLAHE generates a set of pictures of the iris that meet the constraint criterion. The cosine similarity principle is employed in the fitness algorithm to make sure that the images produced belong to the identical category as the original image. The models created by CNN with the best recognition efficiency have accuracy and EER that can approach 95.5 and 0.6809, correspondingly. This demonstrates in full the efficacy of the data augmentation technique [22].

Bhuvaneswari and Devi [23], they introduced the green plan is originally separated from the original retinal picture. The green scheme is next subjected to a standard filter. The WOA methodology is used to select the best scale values after that. subsequently a green plan blocking technique is used to produce an improved image. The result received after using median filtering is taken into consideration in the green plan disguising strategy. Convolutional filtering and Gaussian noise have been applied to this output picture to create an unsharpened green planes picture.

Cai et al. [24], The recommended artificial Physarum swarm mechanism can seek out the best answers through its activities of both growth and decrease as well as information sharing between individuals via self-learning and companion-learning activities. The edges are represented by the Physarum plasmodium in while the transportation nodes are simulated by the outside sources of nourishment. The results show that the proposed system structure can successfully enhance the transportation system’s durability under interruptions while achieving greater efficiency than natural Physarum. Thirdly, a demonstration illustration was developed on the foundation of Mexico City in order to confirm the approach that was suggested.

Cheng et al. [25], introduced the Masked-attention Mask Transformer (Mask2Former), an innovative architecture that can handle any image categorization problem, whether it is panoptic, instance-based, or semantic. Masked attention, one of its essential elements, extracts localized characteristics by limiting cross-attention to expected mask regions. It not only performs significantly better on four well-known datasets than the top specialized designs while requiring at least three times less research work. A new modern facility is particularly achieved by Mask2Former in panoptic identification (57.8 PQ on COCO), example segmentation (50.1 AP on COCO), and semantic division (57.7 mIoU on ADE20K).

Lambourne et al. [26], BRepNet, an architecture for neural networks built to function natively on B-rep information structures, is introduced in this research to obviate the requirement to estimate the model using meshes or clouds of points. Convolutional processors are defined by BRepNet in relation to oriented coedges in the data architecture. A small group of faces, boundaries, and edges can be found in the vicinity of each coedge, and similarities in the vectors of features derived from these components can be found using particular parameters that can be learned.

The abnormal Elallaqi region characteristics were retrieved from aeromagnetic information used in the current study [27]. The Red, Green, and Blue Co-occurrence Matrices (RGBCM) have been applied to the reduced-to-the-pole (RTP) matrix of the Elallaqi district in the South Eastern Desert of Egypt to distinguish texturing from actual aeromagnetic information. The geographical analytical factors used to create the RGBCM convert electromagnetic information into texturing patterns. In this investigation, the textured surface of the RTP grid is analysed using six texture (parameters) characteristics, including correlation, contrary, entropy, uniformity second instance, and variation, from RGB The simultaneous appearance Matrices (RGBCM).

## The proposed framework

Fast pixel-based matching and contour translation techniques are the two suggested approaches that were developed to address the aforementioned issues.

The main goals of the suggested work are listed below.

- Reduce the time it takes to detect a graphic so that you can get more effectively advanced gradient details about it.
- Improve your ability to identify a graphic in an atmosphere of noise and to accurately localize it.
- Create the right edge contours for obligations requiring more complex visual computation.

### Fast pixel based matching

The task of detectors for edges is to locate the limits of objects in images. It is a technique for processing pictures that acknowledges object boundaries. It searches for variations in brightness to function. It is used for the segmentation of images as well as information extracting in disciplines including computer vision, computer image processing, and artificial intelligence. The fuzzy logic, Sobel, Canny, Prewitt, and Robert’s border recognition techniques are commonly used [19–20, 29].

**Fig 1:**
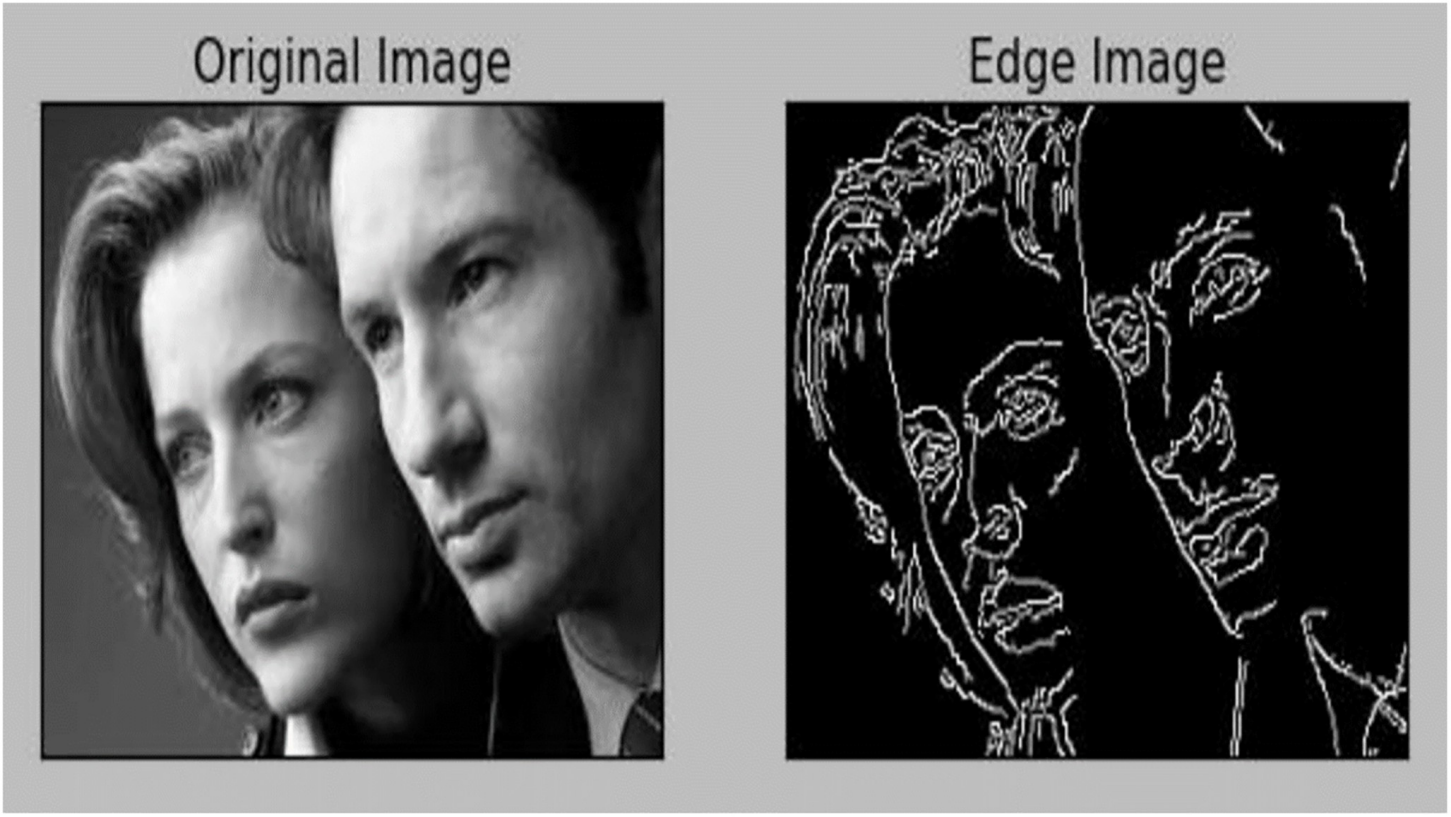
The image segmentation using Canny.

The five procedures that the Canny Edge Detection algorithm takes to find edges are as follows: 1. Easiness Recognising gradients 2. Calculating gradient Third-order suppression 4. Two thresholds 5. Hysteresis-Based Edge Tracking Sliding Noise is a natural part of the images that cameras acquire. since trying to detect boundaries while there is noise can lead to skewed findings. This is depicted in Fig 1.

The procedure used for variety recognition separates pixels in an image that fall inside a given diversity or diversity range. The recognized pixels are then distinguishable from the rest of the image by altering their colour. The mean absolute error is calculated by Eq (1).


$$Mean\;Absolute\;Error\;MAE=\frac{\sum_{i=1}^{M}\sum_{j=1}^{N}I\left(i,j\right)-g\left(i,j\right)}{M}*N$$ (1)

Mean squared error is calculated by Eq (2)

$$Mean\;Square\;Error\;MSE=\sum_{i=1}^{M}\sum_{j=1}^{N}I\left(i,j\right)+f(x,y)-\frac{b}{M}*N$$ (2)

### Active contours-based matching

**Fig 2:**
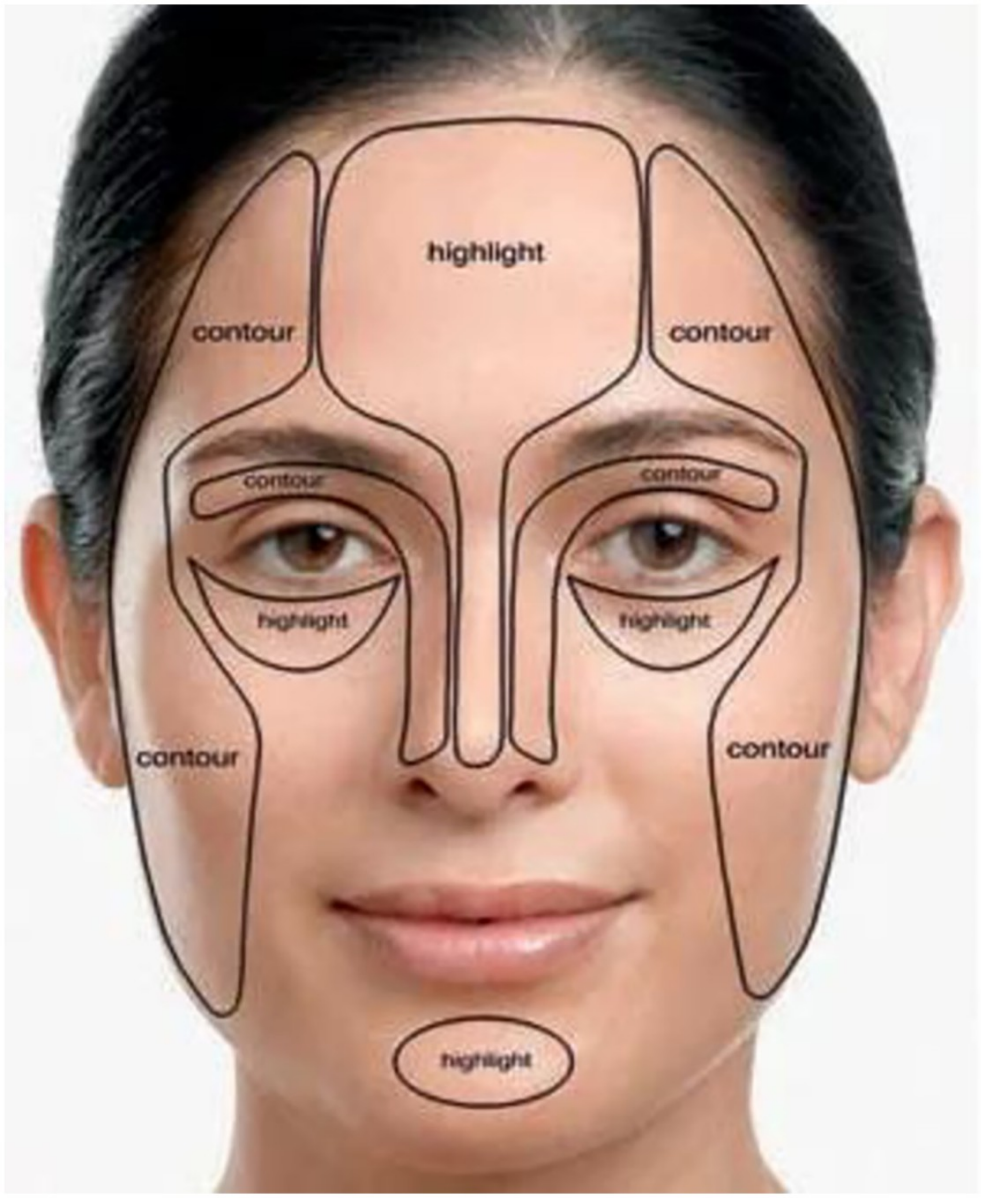
Applying contour on faces (src: http://styletips101.com/wp-content/uploads/2009/04/face-sculpting.jpg).

**Fig 3:**
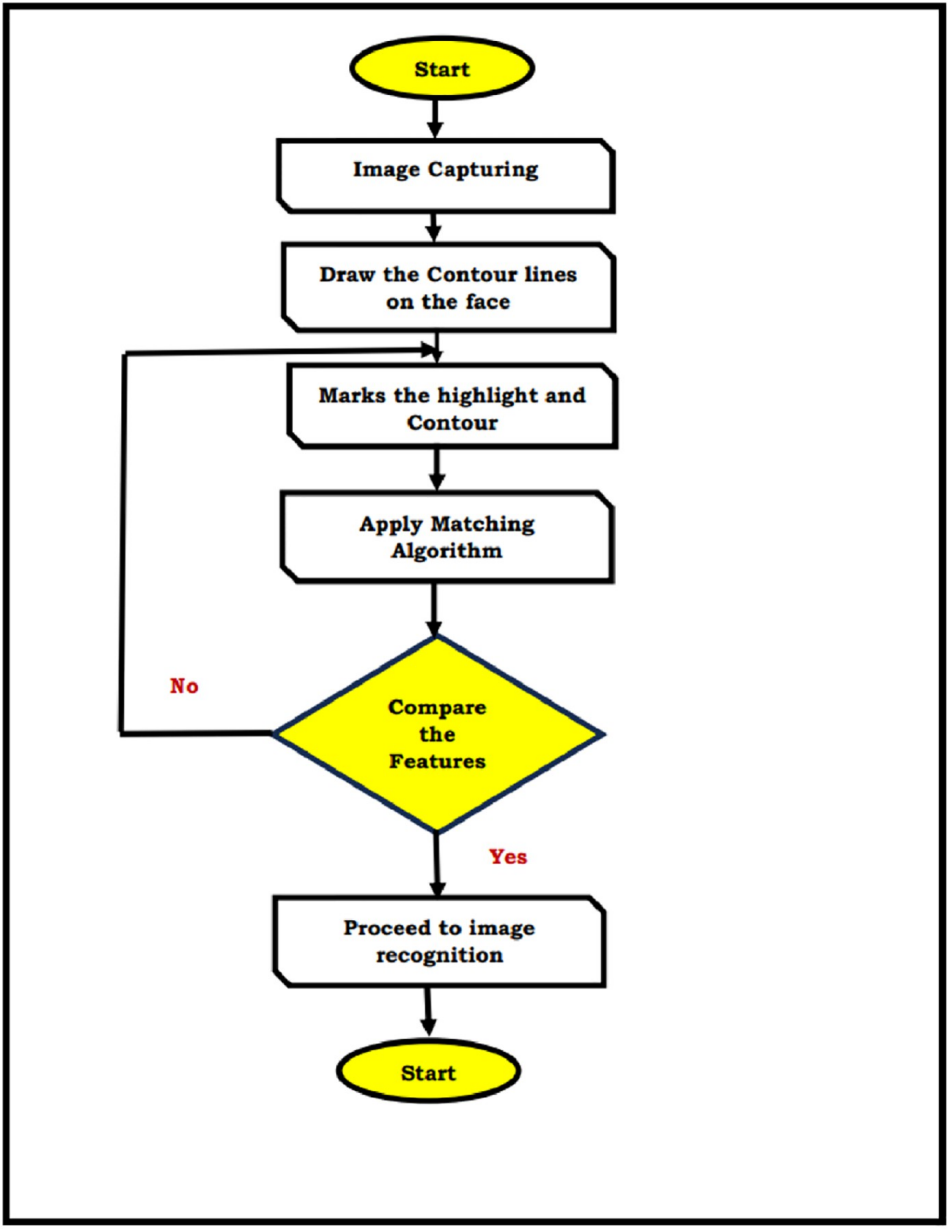
Flow chart for the geometric active contours model.

Dynamic Contour Models (DCM’s) were persistently utilizing in thing, extraction and following. DCM’s were precisely recognizing the stretchable or extend highlights of non-unbending things; as a result of this component, we can give the determined examination of the framework distortion while the humanoid is run all through the whole video movement. In the record, we speak to a human framework extraction and following for step identification utilizing mathematical dynamic form models calculation [10, 11]. The (GAM’s) are especially evolved to dispose of the significant imperfection of the DCM’s that the cycle is generally founded on the beginning bend. Different space locations and a human genome will be gathered by individuals. The design of a running person is removed in that anthropomorphic space identifying by fundamental deductions and phenotypic cycle. The distinctive blueprint actually gets extracted through the GAM’s calculations after these individual elements of layout follows. The bend containment stage and bends displacement phase will be completed in two steps. The primary bend is initially constrained close to the target item by setting the initial position of the current edge from the object’s shape of the previous edge, and it is then poorly defined using the level-set approach [30–32]. Applying forms in face is appeared in Fig 2. The entire face is treated as a shape map, with the territories of steady dark level brilliance encased by the form lines. Dynamic shapes are to characterize smooth shape in the picture and structures shut form for the area [21–24]. Active contour models based on curve flow, curvature, and contour are defined for image segmentation. This model allows for accurate face contour detection in photos with complicated backgrounds, based on both the image boundaries and the previous face form is shown in the Fig 2. The Flow chart for the Geometric Active Contours model is described in Fig 3.

**Matching Algorithm**:

1. Capture image
2. Apply the contours on the face
3. Represent the highlights and contour lines on the face
4. Extract the highlighted features of the face using active geometric contours
5. Match the face with data base using matching algorithm
6. Detect the face

The growing direction of the object limits used to divide a picture into non-overlapping sections is known as an active contour. The zero level-set in active contours represents the curve C, such that

$$C=(x,y)|\varphi (x,y)=0\;of\;a\;level-set\;function\;\phi \left(x,y\right)$$ (3)

if the level-set function φ can be written in the form of

$$\frac{\partial \varphi }{\partial t}+F\left|\nabla \varphi \right|=0$$ (4)

## Results and discussions

### Face detection and tracking

Specifically human eyes are among the simplest features to recognize, face detection algorithms frequently start by looking for them. After that, they look for face landmarks like the mouth, nose, nostrils, and irises. The algorithm does extra tests to verify that it has spotted a face once it determines that it has located a facial region. The algorithms are trained on enormous data sets that contain hundreds of thousands of both positive and negative photos in order to ensure accuracy.

Template matching approach relies on correlating two sets of photos to find a face by comparing them to previously saved examples of common face patterns or traits. This method, though, has trouble dealing with differences in stance, scale, and shape.

Appearance-based technique looks for the pertinent traits in face images using Machine Learning and statistical analysis. Changes in lighting and direction might be challenging for the appearance-based approach. Table 1: shows the Performance indicators of contour base methods with existing methods.

**Table 1:** The efficiency of face detection and tracking by different methods.

S. No	Detection methods	Principle used	Detection Time delay (ms)
1	Knowledge-based	Approaches based on rules that encrypt our understanding of human faces.ˆ	46.4
2	Feature-invariant based	Algorithms that search for a face’s invariant features regardless of the angle or position of the face.	44.8
3	Template matching based	These algorithms contrast the incoming photos with previously recorded face or feature patterns.	36.6
4	Appearance-based	An algorithm that matches templates and learns its pattern database from a set of training photos	31.7
5	Contour based	Illustrated with contour maps	28.8

**Fig 4:**
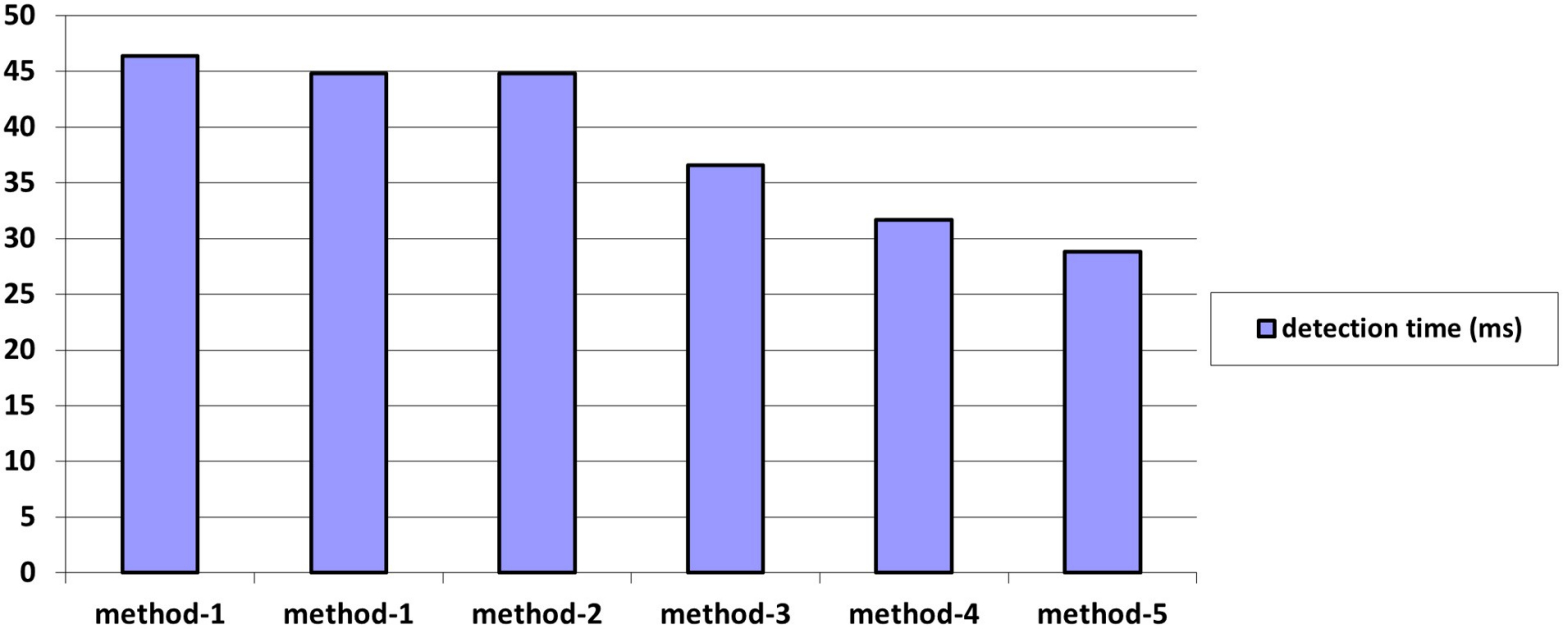
Comparison of various detection methods and time delay (ms).

**Fig 5:**
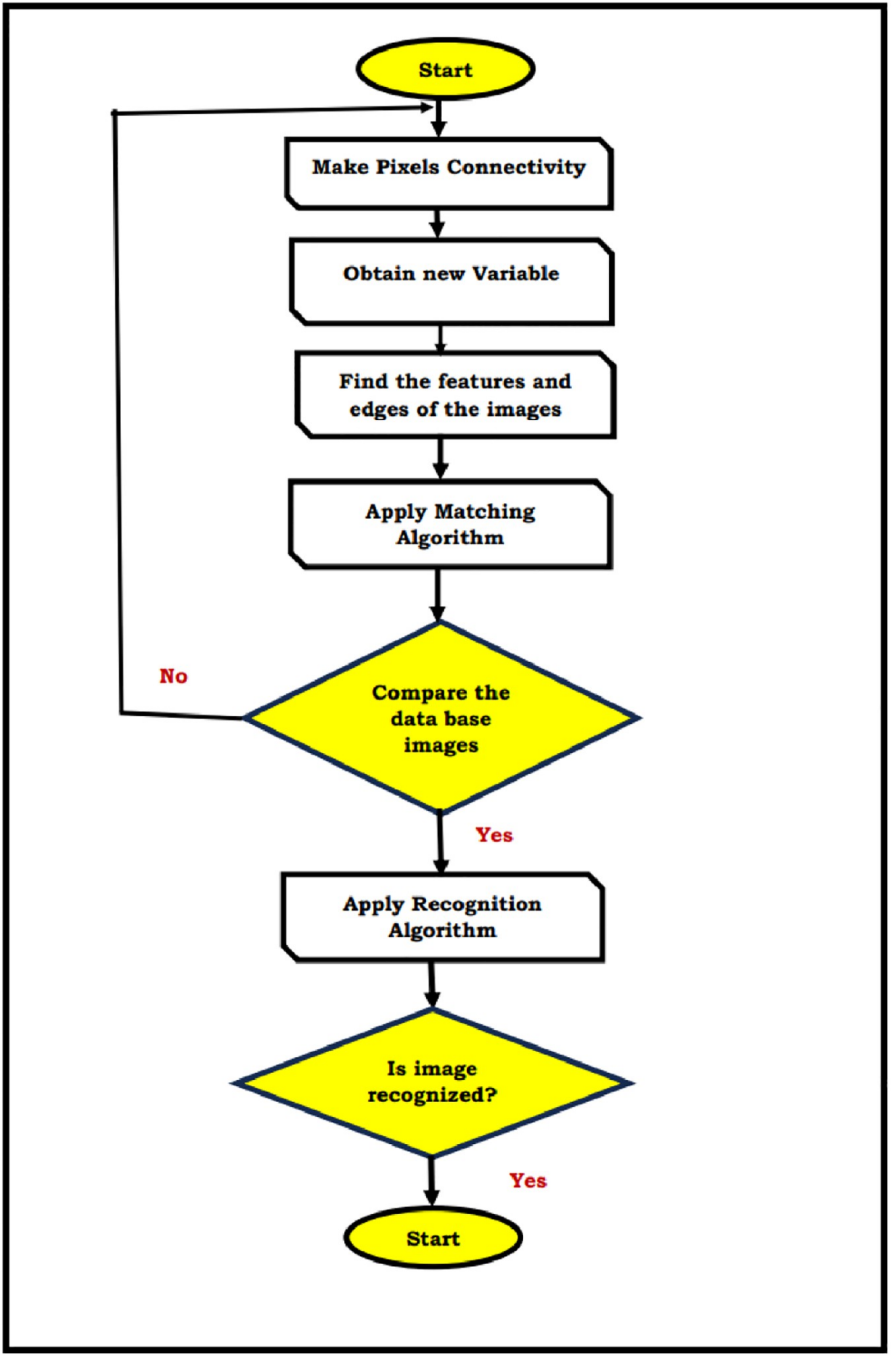
Flow chart for edge detection using connected components operator.

The time-delay analysis of different facial recognition methods and their proposed solution is analyzed in Table 1. From that analysis, we conclude that, the pixel-based algorithms are giving the best solution for edge detection in the images is shown in Fig 4. The Fig 5, clearly explained the edge detection of connected components from the images. It has the different analysis process like matching algorithms, recognition algorithms etc.

#### Face recognition

A statistical method called PCA is used to lower the number of variables in face recognition. The most important information (feature) from the photos (faces) must be extracted. The merits of PCA are Reduces dimensionality of Image, Simple, Fast & Robust. The vectors in the underlying space that discriminate between classes the best is found using linear discriminant analysis (LDA). The Faces were represented as graphs through Elastic Bunch Graph Matching (EBGM), with nodes placed at the Faces’ property points. Table 2 shows that, the face recognition based existing algorithm discussion

**Table 2:** Comparison of various face recognition algorithms.

S. No	Face Recognition Algorithms	Principle used	Detection Probabilities %	Time delay (ms)
1	Principal Component Analysis (PCA)	Using eigen faces	89.13	42
2	Linear Discriminant Analysis (LDA)	Eigenvector-based, supervised linear map	90.2	39.4
3	Elastic Bunch Graph Matching	Using the Fisher face algorithm,	88.5	39
4	The Hidden Markov Model	Set of statistical properties of the signal	91.6	36.6
5	The Multi-Linear Subspace Learning	Using tensor representation	91.8	31.5
6	Neuronal motivate dynamic link matching	The image is represented by layers of neurons and used correlations	92.4	29.5
7	Connected Component Operators (CCO)	Pixel connectivity	94.8	25

**Fig 6:**
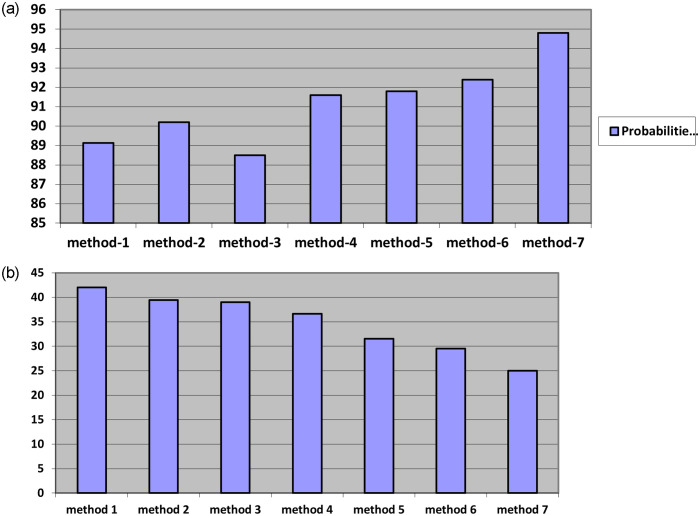
a. Comparison of various Face recognition algorithms vs Detection Probabilities. b. Comparison of various Face recognition algorithms vs Time delay (ms).

The Fig 6(a) & 6(b), illustrate the performances of different face recognition algorithms vs the probabilities of detection from the images and the time delay among the different algorithms in terms of images. Based on the observations, conclude that the connected component operator (CCO) gives better detection probability over other methods. Also, detection time delay is reasonably reduced from other methods. Hence the objectives of the work have been achieved.

**Fig 7:**
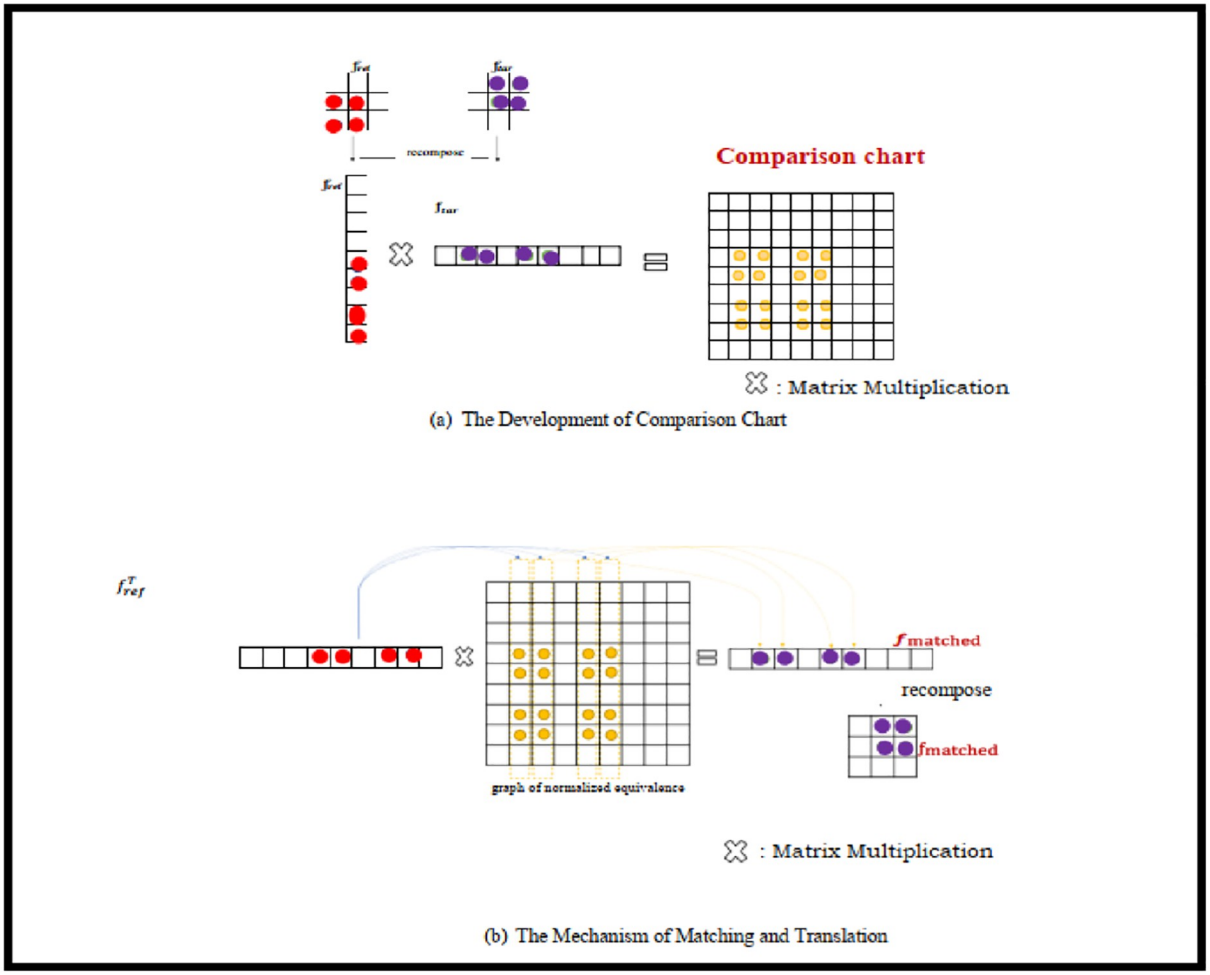
(a) The development of Comparison Chart (eq-5). (b). The Mechanism of Edge Matching and Translation (Eq 6).

**Fig 8:**
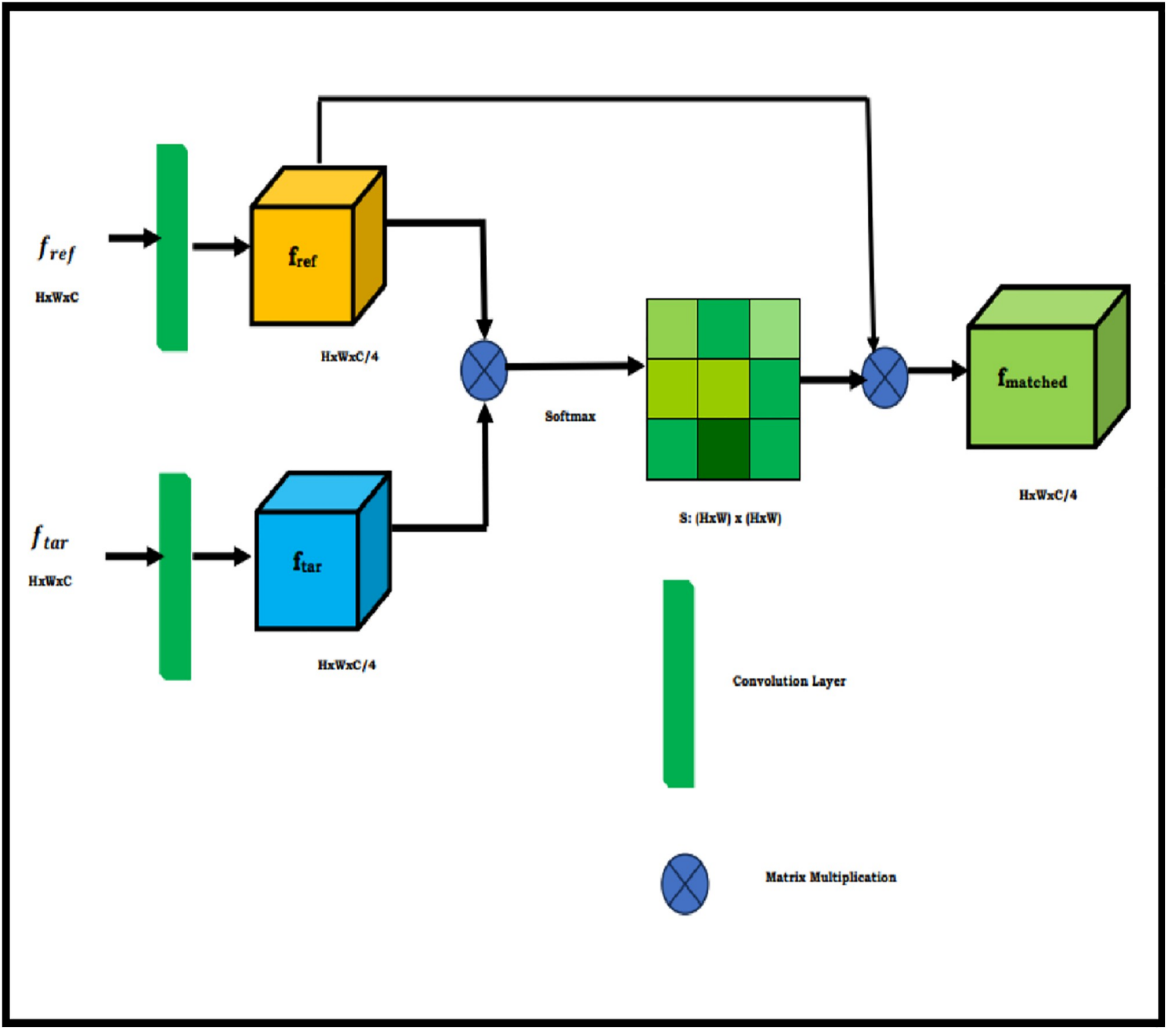
The proposed pixel-matching algorithm process.

The beginning line to the last line: human recognition, foundation Condemnation maps, Detection capacity and furthermore beginning double divisions. The underlying division at the brilliant pixel-goal interface a superior misappraisal, for every location thing. The last picture data is not really disregarded by the better pixels goal. On the off chance that you need very closer picture by next degree of division, we get more data or picture through further switch division and better pixel goal. Table 1 features the upgrades in execution of the proposed calculation. Location productivity improved up to 95.7% is clearly mentioned in Figs 7 and 8.

**Fig 9:**
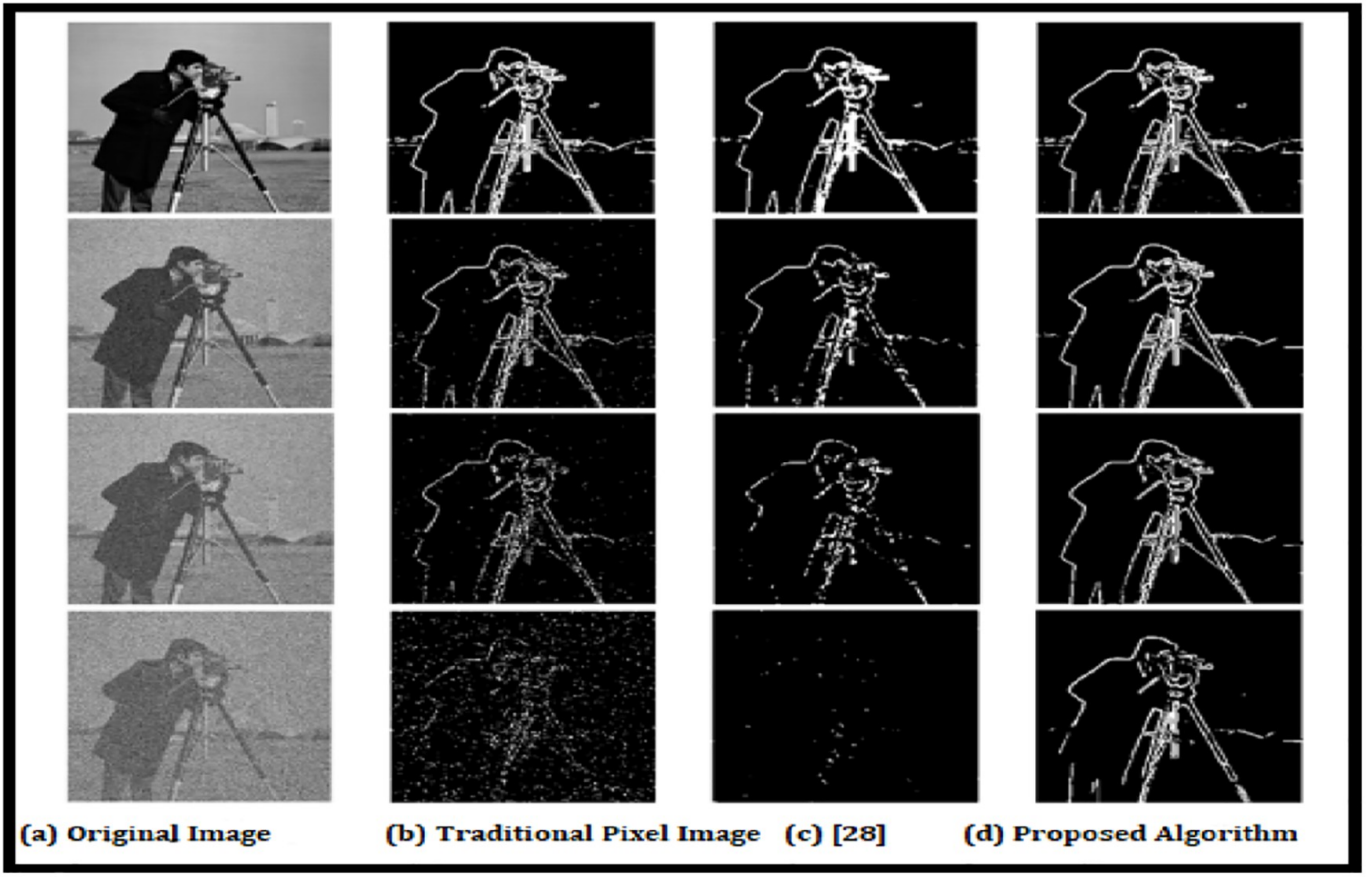
Findings of the photographer recognizing images by the evaluated techniques at various degrees of noise. (a) Experimental Image (b) Detection of algorithm with traditional methods (c) Existing methods results [28] (d) Proposed Algorithm.

In Fig 9(a), the proposed framework is depicted. The parameters that are passed to this module are the feature mappings of the frame that serves as the reference and the objective frame to show, which are represented by the definitions *f*_*ref*_ ∈ *R*^*hxwxc*^ and *f*_*tar*_ ∈ *R*^*hxwxc*^ accordingly, where are the h-height, w-width, and c-channel number. Once characteristic mapping is fed into the component, a 3 X 3 convolutional layer with pad is employed to minimize the channels count of the original characteristic maps from C to C/4, as well as the resultant feature mappings is having dimensions, *f*_*ref*_ ∈ *R*^*NxC*/4^ and *f*_*tar*_ ∈ *R*^*NxC*/4^ correspondingly. This reduces resource utilise and increases the efficiency of our methodology.


$$S=f_{ref}\;f_{tar}^{T}$$ (5)

The resemblance that exists between the i^th^ location on the original feature map and the j^th^ place on the desired characteristic map using S (i; j). Each pixel’s resemblance is determined non-locally, considering all the places in the two feature maps that surround it. Although the inputs come from a sequence of moments, it determines the relationship among two spatial values derived from two temporally framing in the meantime. A significant similarity grade indicates a strong likelihood that two pixels are from an identical surrounding element. In this instance, we can organize the item in addition to matching its look. The completely mismatched attribute map *f*_*matched*_ is subsequently calculated by multiplying the non-local resemblance mapping by the transposition of the decreased referencing characteristic map *f*_*ref*_.


$$f_{matched}=f_{ref}^{T}\;S$$ (6)

**Fig 10:**
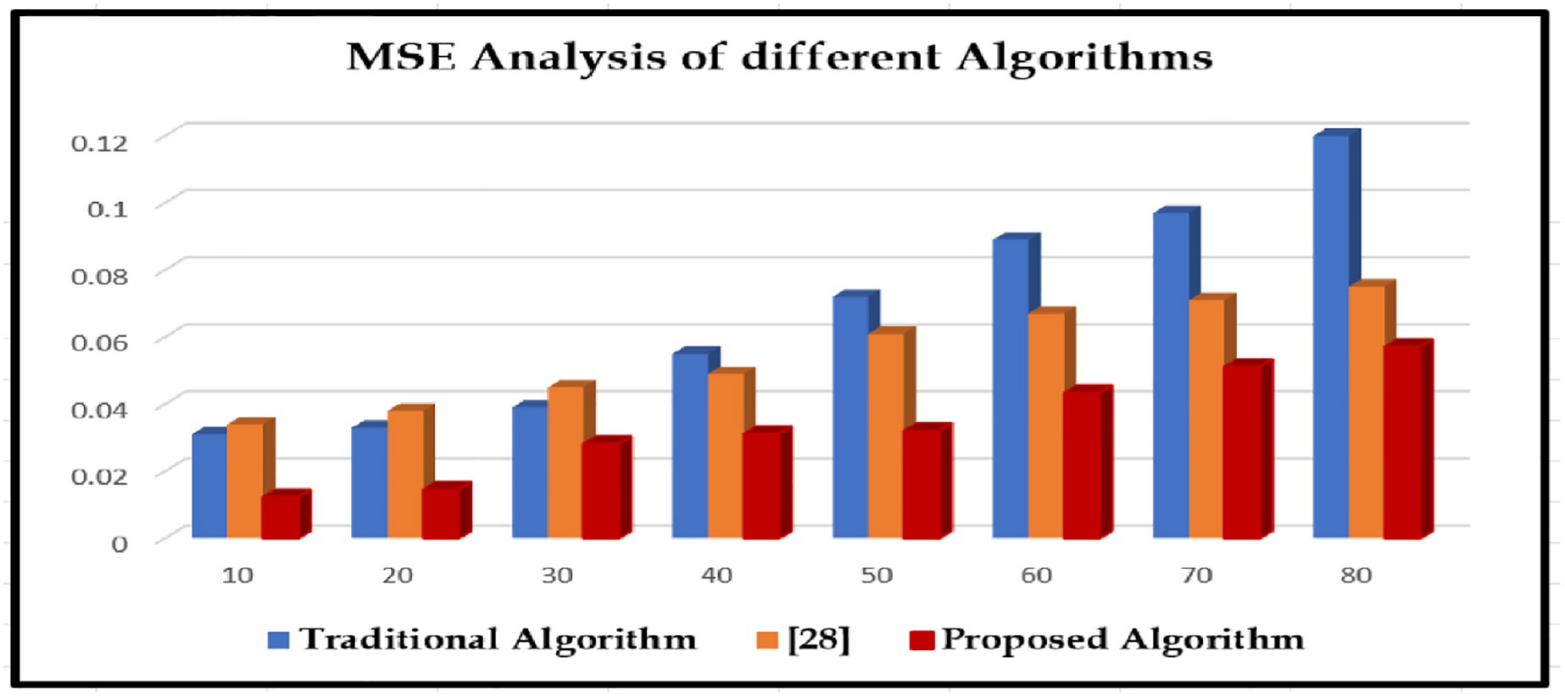
The MSE analysis with different algorithms

**Fig 11:**
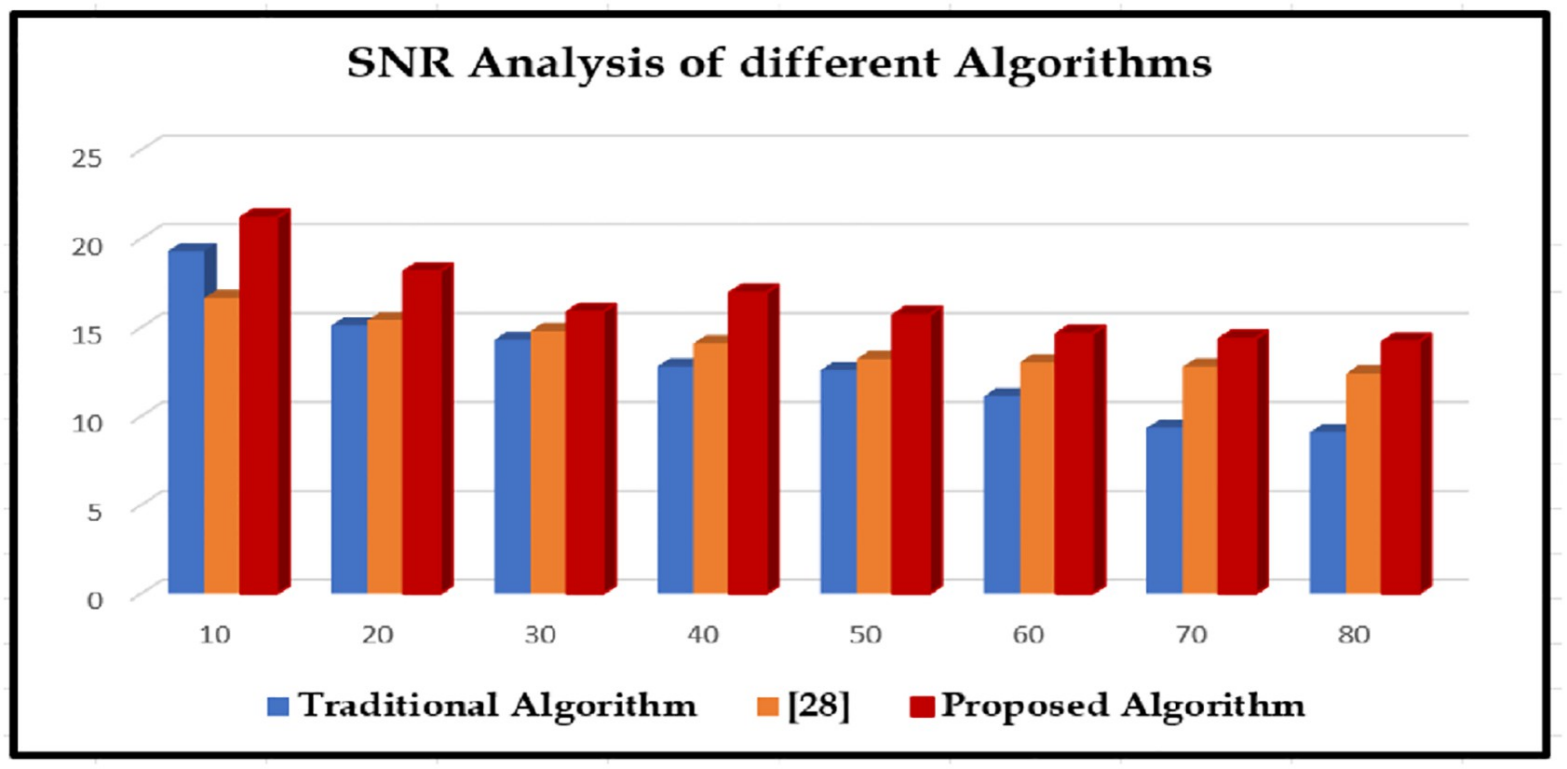
The SNR analysis with different algorithms.

**Fig 12:**
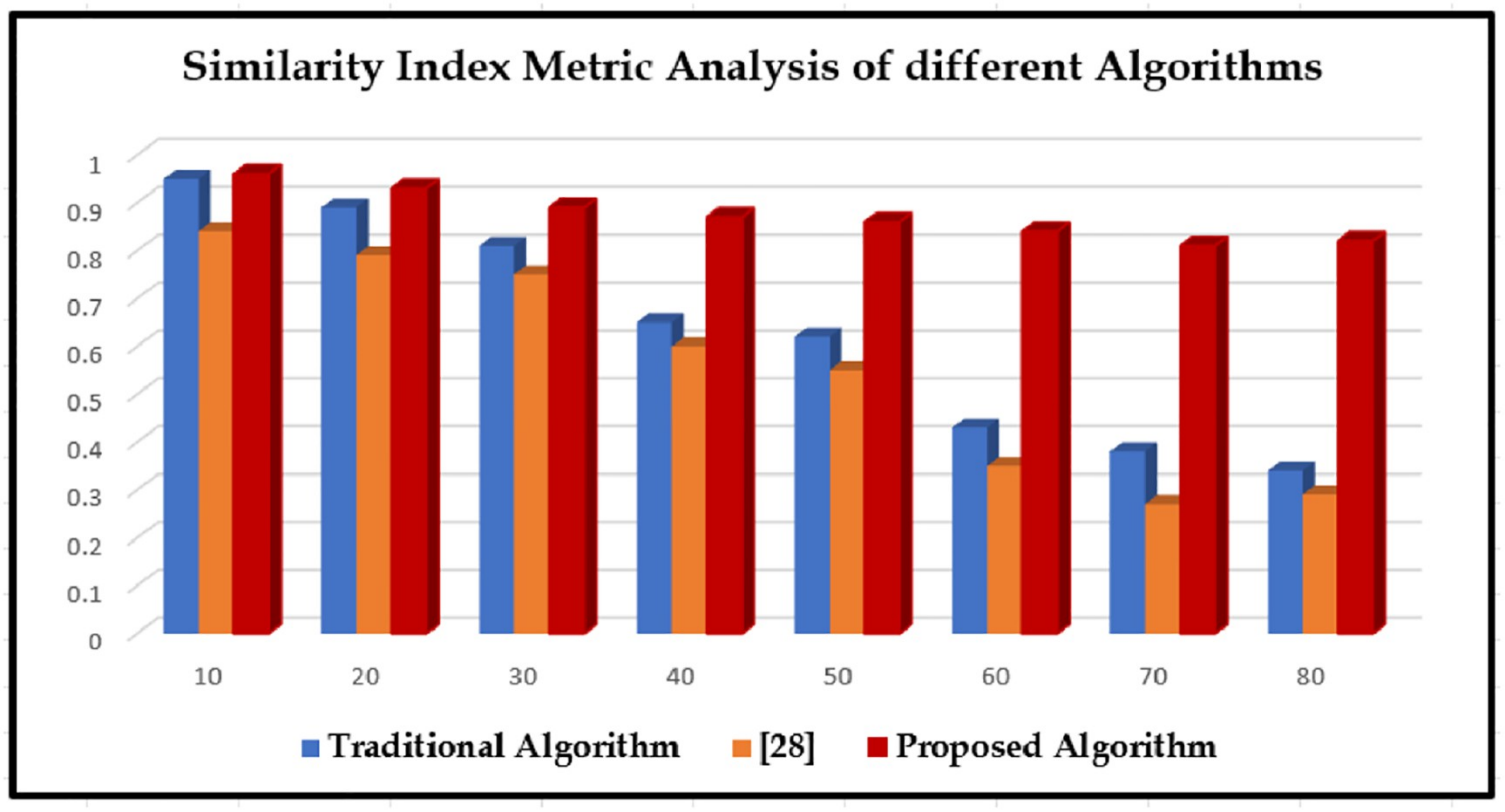
The similarity index analysis with different algorithms.

The proposed matching algorithms is compared with two existing algorithms by measuring the following parameters like mean square error (MSE), peak signal-to-noise ratio (PSNR), and structural similarity index metric (SSIM) represented in (Tables 3–5), and the appropriate line graphical representations are drawn for convenient comparison (Figs 10–12); "sigma" in the plot denotes the standard deviations of included variability and they are depicted in Figs 10–12. The chart shows that, compared to other computations at different noise levels in the study, edges that are recognized by the method developed in this paper acquire lesser MSE, greater PSNR, and higher SSIM numbers. The MSE drops by 0.0124–0.0572, the PSNR improves by 0.610–5.0472 dB, along with the SSIM enhances by 0.0220–0.464.

**Table 3:** The MSE analysis with different algorithms.

Sigma	Traditional Algorithm	[28]	Proposed Algorithm
10	0.031	0.034	0.0124
20	0.033	0.038	0.0146
30	0.039	0.045	0.0282
40	0.055	0.049	0.0312
50	0.072	0.061	0.0321
60	0.089	0.067	0.0434
70	0.097	0.071	0.0512
80	0.12	0.075	0.0572

**Table 4:** The SNR analysis with different algorithms.

Sigma	Traditional Algorithm	[28]	Proposed Algorithm
10	19.31	16.67	21.19
20	15.12	15.41	18.16
30	14.31	14.81	15.87
40	12.81	14.12	16.97
50	12.6	13.24	15.71
60	11.12	13.04	14.65
70	9.35	12.81	14.38
80	9.1	12.42	14.21

**Table 5:** The SIM analysis with different algorithms.

Sigma	Traditional Algorithm	[28]	Proposed Algorithm
10	0.95	0.84	0.96
20	0.89	0.79	0.93
30	0.81	0.75	0.89
40	0.65	0.6	0.87
50	0.62	0.55	0.86
60	0.43	0.35	0.84
70	0.38	0.27	0.81
80	0.34	0.29	0.82

## Conclusion

This developed system assists to reduce the time delay in edge detection. Obtained the improved gradient information and better performance in noise environment. Determine the accurate localization of an image and generate qualified edge contours for higher visual processing tasks. Limitations in traditional edges detection technique is overcome in terms of challenges like illumination changes, position variation, color changes and gesture variation etc. Predominant parameters such as time delay, gradient information, performance in noise, localization, qualified edge contours were improved. It can be effectively utilized in the different initiatives taken by the government to excel the smart and secure life of the people like Digital India, Smart Cities, Innovate India, Manufacturing, Cyber Physical Systems including Artificial Intelligent, IOT and cyber-security etc., Such effective detection system helps to achieve the goal.

In future this works can be extend to reduce the complex computation, noise margin using the Gaussian smoothing and improve the localization, response and Signal to Noise Ratio (SNR).

## Supporting information

S1 File (DOCX)
