# Supplementary material for: Edge detection using fast pixel based matching and contours mapping algorithms
Source: PLoS One. 2023 Aug 11;18(8):e0289823. doi: 10.1371/journal.pone.0289823 (PMC10420379; doi:10.1371/journal.pone.0289823)
Supplement: S1 File — (DOCX) [file pone.0289823.s001.docx]

**Code for- Proposed Edge Detection, calculating MSE, NSR and SIM values**

**import cv2**

**import numpy as np**

**from matplotlib import pyplot as plt**

**img = cv2.imread('chinna.jpg',0)**

**edges = cv2.Canny(img,100,200)**

**plt.subplot(121), plt.imshow(img,cmap = 'gray')**

**plt.title('Original Image'), plt.xticks([]), plt.yticks([])**

**plt.subplot(122), plt.imshow(edges,cmap = 'gray')**

**plt.title('Edge Image'), plt.xticks([]), plt.yticks([])**

**plt.show()**
